# Improved Dispersion of Carbon Nanotubes in Polymers at High Concentrations

**DOI:** 10.3390/nano2040329

**Published:** 2012-10-26

**Authors:** Chao-Xuan Liu, Jin-Woo Choi

**Affiliations:** 1 Department of Electrical and Computer Engineering, Louisiana State University, Baton Rouge, LA 70803, USA; Email: cliu6@lsu.edu; 2 Center for Advanced Microstructures and Devices, Louisiana State University, Baton Rouge, LA 70803, USA

**Keywords:** polymer, carbon nanotubes, nanocomposite, high concentration, dispersion

## Abstract

The polymer nanocomposite used in this work comprises elastomer poly(dimethylsiloxane) (PDMS) as a polymer matrix and multi-walled carbon nanotubes (MWCNTs) as a conductive nanofiller. To achieve uniform distribution of carbon nanotubes within the polymer, an optimized dispersion process was developed, featuring a strong organic solvent—chloroform, which dissolved PDMS base polymer easily and allowed high quality dispersion of MWCNTs. At concentrations as high as 9 wt.%, MWCNTs were dispersed uniformly through the polymer matrix, which presented a major improvement over prior techniques. The dispersion procedure was optimized via extended experimentation, which is discussed in detail.

## 1. Introduction

Polymers possess a great variety of material characteristics (e.g., mechanical flexibility, optical transparency, biocompatibility, chemical stability, *etc.*) enabling them to be used in diverse applications such as microfluidic systems and bio-implantable systems. A polymer can be produced in huge volumes thanks to the development in its manufacturing industry. For example, microelectromechanical systems (MEMS) can be readily made from polymer by processes such as cast molding, injection molding, hot embossing and photolithography.

However, due to lack of electrical conductivity in most polymers, the role of this material has been limited to a structural component in most applications. Often, polymer-based MEMS devices require conductive elements to electrically control or collect signals from systems. While metal suffers coherent incompatibility issues with polymers, a mixture of polymer and nanoscaled fillers—termed nanocomposite provides an alternative of incorporating conductivity into polymer systems. This class of material is unique in the sense that it retains many desirable features of polymers (flexibility, biocompatibility, processability) yet adds electrical conductivity and/or piezoresistivity from the nanofiller which is not an intrinsic property of most polymers. By utilizing polymer nanocomposite, components such as conductive electrodes and sensor elements could be incorporated into conformal all-polymer systems.

Polymer nanocomposite is composed of a thorough mixture of polymer matrix and nanoscale filling materials. To use as the matrix of nanocomposite, there are numerous polymers of diverse properties to choose from, including both plastics and elastomers which are the main two types of polymers. For the purpose of sensing specifically, a good deal of research effort has been committed to select an appropriate hosting matrix for the nanocomposite. Various polymers such as poly(methyl methacrylate) (PMMA) [1,2], polycarbonate (PC) [3], poly(ethylene) (PE) [4], poly(L-lactide) (PLLA) [5], *etc.* have been incorporated with nanofillers to construct strain sensors, which are capable of holding larger tensile strain than conventional metallic strain gauges [6].

Compared with above polymers, silicone-based elastomer poly(dimethylsiloxane) PDMS owns superior mechanical elasticity as it easily holds over 100% of tensile strain without any structural failure [7], making it an ideal choice for large-range strain sensing applications. Its flexibility allows it to be readily attached to curved surfaces, which is often necessary in biomedical sensors. Moreover, PDMS being a chemically inert and biocompatible material is widely used in microfluidics and biomedical areas [8].

Since MEMS-based sensors require feature sizes on the microscale, conductive fillers of polymer nanocomposite typically have feature size on the nanometer scale, so that the conformity of microstructures could be ensured. Common nanofillers include carbon nanotubes, carbon black (nanoscaled carbon particles), metal particles and flakes [9]. Amongst these, carbon nanotubes are particularly interesting candidate for sensing applications.

Ever since the discovery of carbon nanotubes by Iijima in 1991 [10], numerous research has been conducted to explore the potentials of this “magic” material [11]. With a high aspect ratio because of its long tubular structure, carbon nanotubes demonstrate relatively high electrical conductivity. For example, compared with other conductive nanofillers (e.g., metal flakes or particles with lower aspect ratios), carbon nanotubes composites of various polymers reach percolation threshold at a lower weight percentage [9,12]. Probably, tubular structures allow the formation of a more efficient electron-conducting network in CNT-based composite. Also, the piezoresistivity of CNTs make them a suitable candidate for sensors: when a CNT-based composite is exposed to mechanical deformation, the geometry and interconnections of nanotubes within the polymer matrix vary accordingly, which leads to a change in its electrical resistance. Further, CNTs are one of strongest materials known to man, with tensile strength up to 63 GPa using multi-walled carbon nanotubes (MWCNTs) [13], giving them another edge over other materials for fabricating robust sensors.

In this work, multi-walled carbon nanotubes were chosen over single-walled carbon nanotubes (SWCNTs) because MWCNTs generally offer better conductivity [32]. Plus, economic wise MWCNTs generally cost less to purchase. Thus, MWCNTs were picked as the preferred material over SWCNTs [14].

## 2. Results and Discussion

In itself, dispersion is a spatial property whereby the individual carbon nanotubes are spread with the roughly uniform number density throughout the continuous polymer matrix. The first challenge is to separate the tubes from their initial aggregated assemblies, which is usually achieved by local shear forces. Direct manual mixing of CNTs with polymer resin, though the simplest approach, does not create sufficient local shear force and therefore leads to poor dispersion of CNTs inside polymer matrix.

More effective separation of CNT bundles requires the overcoming of the inter-tube van der Waals attraction [15]. Depending on the tube shape/size and the orientation of nanotubes with respect to each other, such an attraction can act within a spacing of a few nanometers [16]. For closely packed tubes within a medium, the surface adsorption of a dispersant, or the wetting of the polymer/solvents, both require a temporary exfoliation state. Physical approaches such as shear mixing [17], mechanical stirring, sonication [18], ball milling [19] and micro-bead milling [20] processes have been employed for this purpose. Although these techniques may appear very different, they are all governed by the transfer of physical shear stress onto nanotubes which breaks down bundles.

In shear mixing, for example, the separation of individual CNTs from bundles is achieved in shear flow induced by the rotation of an extrusion in a polymer solution or melt. Usually, dispersion via shear mixing is only achievable for specific types of MWCNTs, with a high shear rate in a rather viscous medium. Terentjev *et al.* demonstrated that nanocomposite containing high loading concentrations of CNTs (up to 7 wt.%) could be dispersed via this technique [21]. However, the processing time significantly goes up as loading concentration rises. More importantly, shear mixing tends to section carbon nanotubes into shorter length scale, thereby reducing their conductivity significantly—an undesired attribute for nanocomposite intended for use as a sensor material.

The dispersion of carbon nanotubes could be assisted by the introduction of a common solvent—an organic solution which dissolves polymer resin easily and at the same time allows monodispersion of carbon nanotubes. In this case, two dispensed solutions sharing common solvent but containing carbon nanotubes and polymer resin respectively, undergoes mechanical stirring or the increasingly popular sonication process. Following that, two solutions are mixed together to further go through stirring or sonication. Finally with the complete evaporation of solvent, CNTs would leave dispersed in polymer. Here the choice of organic solvent is critical for determining the final dispersion quality and depends on the polymer matrix. For PDMS alone, various organic solvents have been reported to assist dispersion of CNTs, such as toluene [22], tetrahydrofuran (THF) [23], chloroform [24,25], dimethylformamide (DMF) [26], *etc.* While each report claims high dispersion quality of CNTs, there is lack of standard characterization protocol for dispersion of CNTs within polymers [15], leaving room for subjective judgment. Comparative experimental tests are still needed to verify optimal solvent choice for the dispersion of carbon nanotubes.

It should be noted that even with optimal common solvent to help dispersion, the optimization of process conditions is still critical to ensure final dispersion quality. In the stage of solvent evaporation, for instance, as CNTs concentration continuously increases so does the re-aggregation effect of CNT bundles. Thus, this step needs to be best shortened to minimize the compromise of dispersion.

### 2.1. Materials and Reagents

Pristine multi-walled carbon nanotubes (MWCNTs) used in this work were purchased from Cheaptubes, Inc., (Brattleboro, VT, USA) with a relative purity >95 wt.%. The dimension of the MWCNTs was 20–30 nm in outer diameter, 5–10 nm in inner diameter and 10–30 µm in length. To comparatively study the effect of surface functionalization on its dispersion state, MWCNTs treated with carboxylic acid groups (MWCNTs-COOH) were also obtained from the same company (MWCNTs-COOH contains 1.23 wt.%-COOH groups. The other properties remained the same, with relative purity >95 wt.%, 20–30 nm in outer diameter, 5–10 nm in inner diameter and 10–30 µm in length, to affirm the validity of the comparative study.

The polymer matrix used in this work—poly(dimethylsiloxane) (PDMS)—is a silicone elastomer. Specifically, Sylgard 184 silicone elastomer kit was purchased from Dow Corning Inc. (Midland, MI, USA), which had two parts: polymer base resin and curing agent. The two parts are recommended to be mixed at the ratio of 10:1 and exposed to thermal curing in order to realize solidified PDMS. In fact, the mixing ratio could be varied in order to tune mechanical properties of PDMS (Young’s modulus), making it a versatile material to utilize.

### 2.2. Selection of Optimal Solvent for the Dispersion of CNTs within PDMS

The dispersion assisting solvent depends on the type of polymer and CNTs, as it needs to dissolve both well to be effective. Also, even when a solvent disperses both polymer and filler well separately, the combination of the two could have an adverse effect on the dispersion state. Therefore, the conclusion of an optimal common solvent could only be drawn after careful comparative experimental studies.

#### 2.2.1. CNTs Dispersion in Different Organic Solvents

As noted earlier, a variety of organic solvents have been used to assist dispersion of CNTs in polymers. Solvents including toluene [22], tetrahydrofuran (THF) [23], chloroform [24,25], dimethylformamide (DMF) [26], *etc.*, in particular, have been reported to reach great dispersion. Nevertheless, due to the lack of sufficient dispersion characterization data from these reports, experimental studies are still required to compare their actual performances and verify optimal choice for the dispersion of carbon nanotubes. An advantage of comparative study is that only relative dispersion quality is required to be evaluated, based on which a best solvent could be chosen from the solutions tested.

To compare the dispersion of MWCNTs in different solvents, four organic solutions including toluene, chloroform, DMF, and THF, as shown in Table 1, were used for experimental test in the following manner.

**Table 1:** Important properties of experimentally tested organic solvents.

Organic solvent	Chemical formula	Density (g/mL @ 20 °C)	Boling point (°C)	Vapor pressure (kPa @ 20 °C)
Toluene	C_6_H_5_CH_3_	0.86	110.6	2.93
Chloroform	CHCl_3_	1.48	61.2	21.1
Tetrahydrofuran	C_4_H_8_O	0.89	66	19.3
Dimethylformamide	C_3_H_7_NO	0.94	153	0.3
**PDMS base resin**	**(C_2_H_6_OSi)**	**1.11**	**N/A**	**N/A**

**Figure 1:**
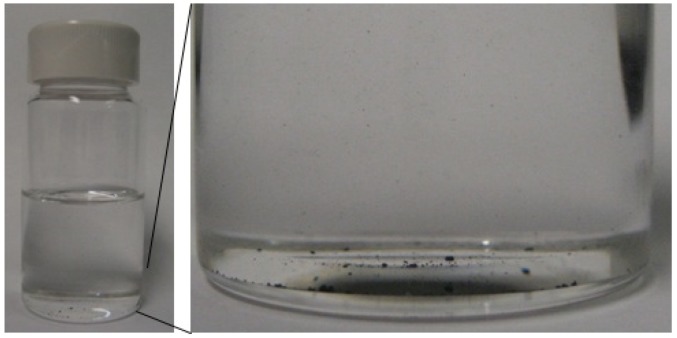
Images showing 3 mg of multi-walled carbon nanotubes (MWCNTs) being added into 10 mL of chloroform. In the magnified view black bundles settled on vial bottom were from as-is MWCNTs.

First, quantities of pristine MWCNTs weighing about 3 mg were added into four vials containing 10 mL of respective solutions, yielding a concentration of 0.3 mg/mL, as shown in Figure 1 (chloroform).

Here, a low concentration was desired to offer partial optical transparency in dispersed solutions. Through trial and error, it was found that solution with concentrations higher than about 1 mg/mL, after dispersion, would become completely nontransparent, which was not desirable for direct visual observation. On the other hand, it was found in experiments that a concentration of 0.3 mg/mL or lower was possible for direct visual observation of dispersion qualities. Although the 0.3 mg/mL threshold was somewhat empirical, MWCNTs dispersed around this concentration were able to be observed clearly and consistently. With this small amount of MWCNTs the inaccuracy of the weight equipment was relatively significant (±1 mg) which might cause variations in the concentration of MWCNTs in different solvents. However, later optical observation would prove, though, this variation was not significant in affecting the dispersion quality of solutions.

**Figure 2:**
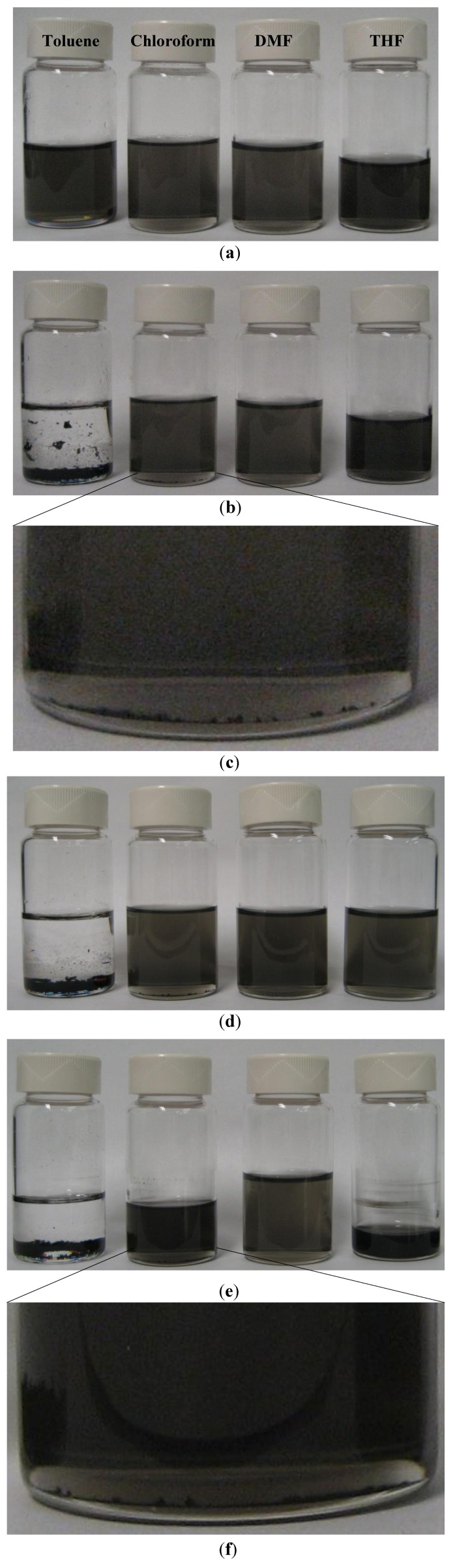
MWCNTs dispersed in different organic solvents via 30 min of sonication. Solutions from left to right: toluene (0.3 mg/mL MWCNTs), chloroform (0.3 mg/mL), dimethylformamide (DMF) (0.3 mg/mL), tetrahydrofuran (THF) (0.4 mg/mL). (**a**) Dispersion state directly after sonication, showing no visible MWCNT bundles; (**b**) Solutions at 70 h after sonication showing reaggregation effect of MWCNTs in order of toluene >> chloroform > THF > DMF; (**c**) Magnified view of visible MWCNTs bundles in chloroform solution; (**d**) one week after sonication. Volume of THF solution was slightly adjusted to match the others after 4 days with no further sonication; (**e**) eight months after sonication. Solutions have evaporated to different extents but three out of four dispersions remained stable; (**f**) Magnified view of visible MWCNTs bundles in chloroform dispersion, indicating that amount of bundles remained about the same with (**c**).

After introducing MWCNTs into four solutions, each mixture was then sonicated using a mild sonication bath (FS20D Fisher Scientific, frequency 42 kHz, output power 70 W) for 30 min at nominal power. This process yielded semi-transparent dispersed MWCNT suspensions, as shown in Figure 2a, composed of individual nanotubes and micro-sized bundles which were invisible to the bare eye.

The stability of CNT dispersion state is a valuable indication of its dispersion quality, as CNTs tend to reaggregate into bundles with time in an unstable environment. The longer dispersion lasts and the fewer/smaller CNT bundles occur, the higher its dispersion quality. Here a simple approach of optical observation of CNT bundles was applied to evaluate dispersion quality. Although this approach could not determine the absolute quality of dispersion, it was well suited for comparative studies, where only relative information was extracted and compared from samples tested under identical conditions.

All four vials were held still after their sonication. Seventy hours later, unstable dispersions showed signs of reaggregation to different extents, as indicated in Figure 2b. Visible MWCNT bundles were observed to settle at the bottom of vials, as shown in Figure 2c (chloroform) as an example. In the worst case of toluene, suspension showed apparent phase separation, with MWCNTs almost completely settled at the bottom and upper solution void of dispersed MWCNTs. Based on visual observation of the amount of reaggregated MWCNTs in various comparative experiment settings, it was likely that the reaggregation effect had an order of: toluene > chloroform > THF > DMF.

One week after sonication, as shown in Figure 2d, solutions largely remained their dispersion state, other than the now transparent toluene solution. Figure 2e shows that, even after an extended holding period of 8 months, MWCNTs dispersion in the other three solutions maintained its stability, regardless of the fact that part of solutions evaporated causing CNT concentration to rise. In the case of chloroform, especially, the amount of its CNT bundles, as shown in Figure 2f, did not increase notably from the 70 h mark, which proved it to be also a stable dispersion. Overall, despite minor variations in dispersibility of MWCNTs, all three solvents including chloroform, THF and DMF could be considered candidates to help the dispersion of CNTs inside polymer nanocomposite.

#### 2.2.2. Solubility of PDMS Base Polymer in Different Organic Solvents

As a common solvent to assist dispersion of polymer nanocomposite, another important requirement is its ability to dissolve the polymer matrix. Therefore, the choice of solvent varies significantly depending on the type of polymer matrix. For PDMS specifically, the four organic solvents (toluene, chloroform, DMF, THF) which have been used in previous reports were used here for testing.

It should be noted that since PDMS is a two-part thermal curable polymer, only one part should be used in the dispersion process of CNTs. As dispersion usually takes more than a few hours, the presence of two parts mixed together could render PDMS partially polymerized, which then would not be usable anymore for microfabrication. With a manufacturer-recommended mixing ratio of 10:1, PDMS base polymer resin occupies more than 90% weight of the polymer matrix, thus the base polymer is generally the part used for dispersion with CNTs. Curing agent, on the other hand, would be added after the evaporation of the common solvent, which will be further discussed later.

Out of the four organic solutions tested, toluene was found to have great solubility for PDMS base polymer. Nonetheless, it could not be an ideal candidate as common solvent because of its relatively poor dispersion of MWCNTs.

DMF, although had the best dispersibility for MWCNTs, was found to react with PDMS base resin. Upon mixing of these two solutions, a white colored gel-like substance was formed due to chemical reaction. Thus, while it may be useful to be used for dispersion of other polymer matrices, in the case of PDMS, due to chemical incompatibility, it would not be useful for the fabrication of nanocomposite. 

Interestingly, chloroform and THF were both found to own high solubility of PDMS base resin, able to dissolve PDMS at concentration higher than 0.3 g/mL. Because of their relatively high dispersion of MWCNTs, both of these could potentially work as common solvents for the preparation of polymer nanocomposite. Further tests were conducted (as follows) as visual observation alone may not be sufficient to distinguish which of the two solutions would work better for PDMS.

#### 2.2.3. The Effect of PDMS on Dispersed Carbon Nanotubes in Different Organic Solvents

Supposedly, as long as the common solvent can dissolve MWCNTs and PDMS well separately, it should work for the combination of the two. The two suspended solutions could simply be poured together to go through further sonication in order to achieve high quality dispersion. Surprisingly, that was found to be not the case with certain organic solvents.

From the above section, both THF and chloroform were promising candidates to work as common solvent due to their exceptional ability to disperse MWCNTs and PDMS separately. However, when MWCNTs and PDMS were both present in the solvent, THF and chloroform had dramatically different performances.

**Figure 3:**
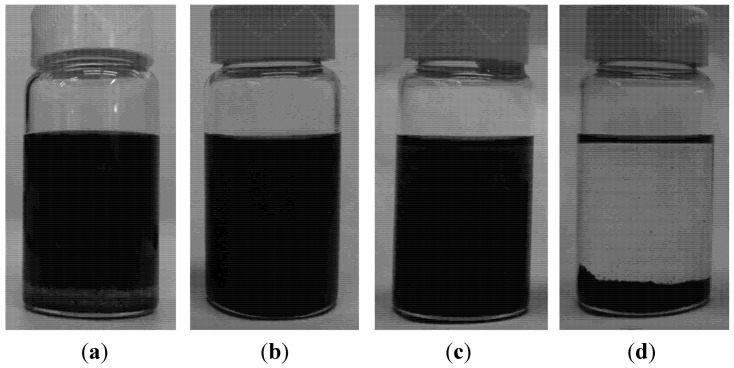
Vial images showing effect of poly(dimethylsiloxane) (PDMS) on dispersion state of MWCNTs in THF solution: (**a**) PDMS added into dispersed MWCNTs-THF solution; (**b**) THF solution containing PDMS and MWCNTs directly after 30 min of sonication; (**c**) solution at 30 min after completion of sonication, showing visible CNTs bundles, and (**d**) solution at 21 h after sonication, showing complete phase separation which indicated the instability of dispersion.

In the case of THF, firstly MWCNTs were dispersed at 0.4 mg/mL (±0.2 mg/mL) via sonication for 10 min. Then, PDMS base resin at 0.15 g/mL concentration was added into the already-dispersed CNTs, as shown in Figure 3a. From Table 1, as the density of PDMS base resin (1.1 g/mL) was higher than THF (0.89 g/mL), PDMS settled at the vial bottom and could be clearly told from the dispersed CNTs. Afterwards, the mixture was sonicated for an additional 30 min, resulting in a fully dispensed solution, as in Figure 3b. However, this dispersion state was not stable with time. MWCNTs almost started reaggregating immediately, forming visible bundles just 30 min after sonication was finished, as seen in Figure 3c. Moreover, after a period of 21 h, originally dispersed MWCNTs had now completely settled at the vial bottom, as shown in Figure 3d, leaving the upper portion of solution fully transparent.

In fabrication of polymer nanocomposite, PDMS base was normally first dissolved in an organic solvent before being added into a CNT dispersion solution, instead of directly being added like in the above process. With the pre-dissolution of PDMS, similar effects also occurred when MWCNT dispersion significantly deteriorated after the introduction of PDMS content. The reason for the adverse effect of PDMS on THF-CNT dispersion has yet to be understood, however, although THF did not alter properties of PDMS base resin when mixed with it, some functional groups on PDMS base molecules could have affected the affinity between THF and MWCNTs.

**Figure 4:**
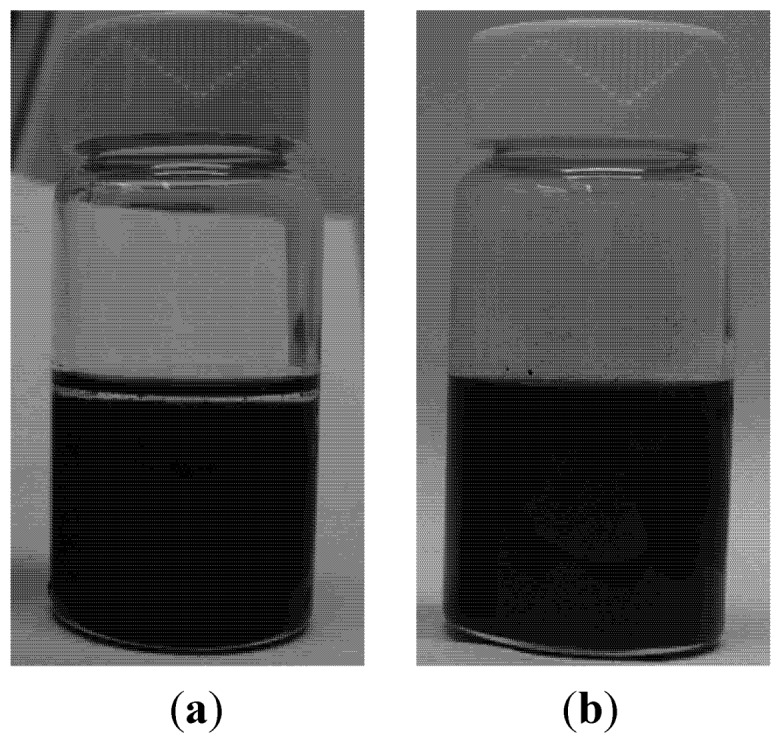
Vial images showing effect of PDMS on dispersion state of MWCNTs in chloroform solution: (**a**) PDMS added into dispersed MWCNTs-chloroform solution, staying on top portion and (**b**) Chloroform solution containing PDMS and MWCNTs 42 h after sonication.

In the case of chloroform, similar experimental procedures were carried out to test the effect of PDMS on the dispersion state of MWCNTs. Briefly, MWCNTs were first dispersed at 0.4 mg/mL (±0.2 mg/mL) via sonication for 10 min. Then, PDMS base at around 0.12 g/mL concentration was added into solution, as shown in Figure 4a. Since the density of PDMS base (1.1 g/mL) was lower than chloroform (1.48 g/mL), PDMS stayed at the solution top separated from CNT. Then, solution was mechanically stirred for 14 min (to help expedite dissolution of PDMS) and consequently sonicated for 1 min, leading to a fully dispensed solution. Unlike THF-CNT dispersion, the chloroform suspension remained much more stable, as shown in Figure 4b, showing no visible deterioration of dispersion state even at 42 h after sonication.

Based on the above visual comparison in Figure 4 of THF and chloroform in which PDMS and MWCNTs were dispersed, it can be concluded that, with similar circumstances, chloroform yielded a much more stable—thus higher quality dispersion of PDMS base and MWCNTs. Therefore, the best choice of common solvent among tested solutions should be chloroform.

### 2.3. Effect of CNT Functionalization on Dispersion

**Figure 5:**
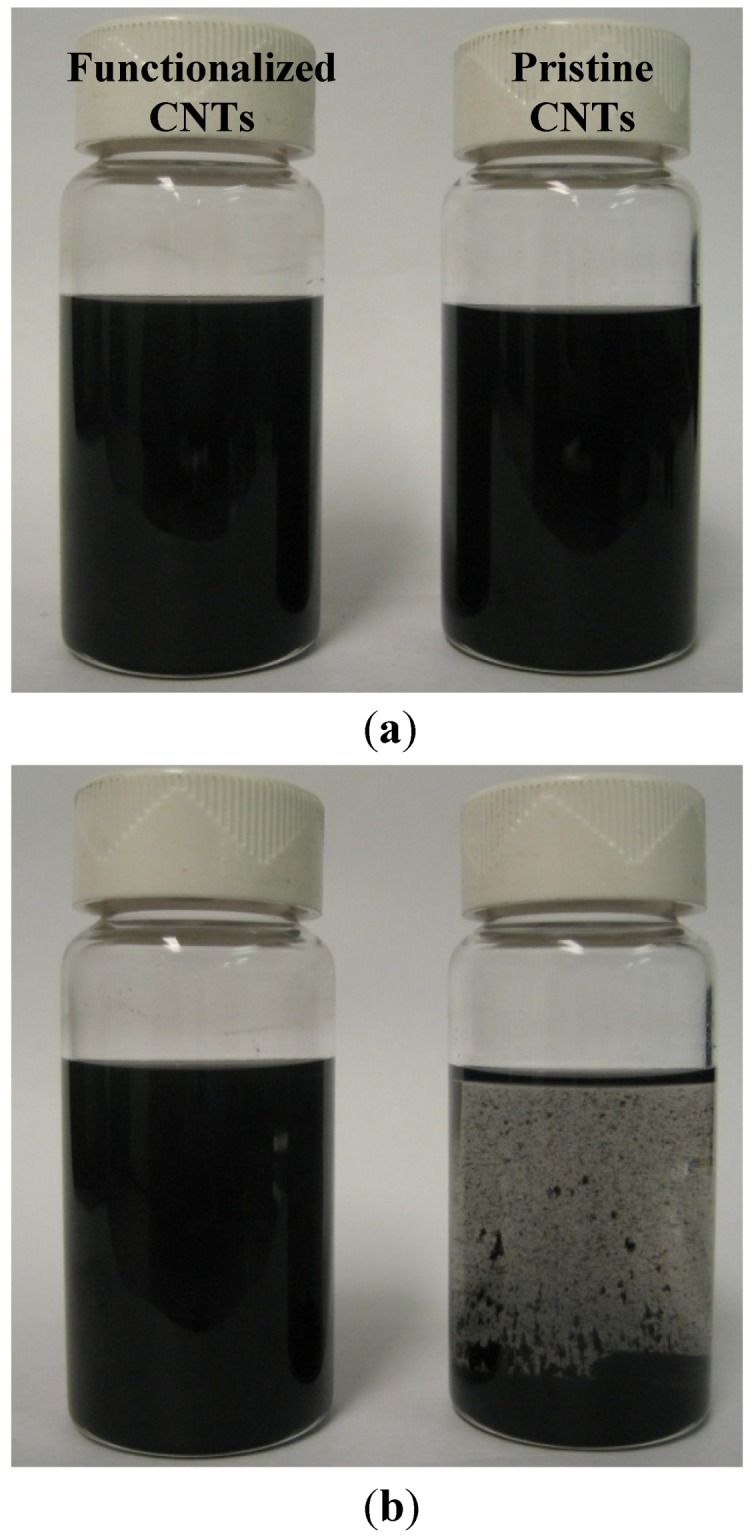
Comparative studies of functionalized and pristine MWCNTs in their dispersion with polymer matrix in common solvent: (**a**) initial dispersion of MWCNTs directly after sonication and stirring showing nontransparent solutions and (**b**) dispersion at 4 h after the stoppage of stirring, with pristine MWCNT case showing clear phase separation.

It has been reported that carbon nanotubes with functionalized surfaces by carboxyl (–COOH) groups, compared to their pristine counterpart, could have better dispersion in polymer matrices [22,27]. To verify the effect of surface functionalization, comparative experiment was carried out, in which pristine and –COOH carbon nanotubes were dispersed with PDMS base in chloroform solutions in a parallel fashion under similar conditions. Briefly, both pristine MWCNTs (0.14 mg/mL) and COOH-MWCNTs (0.16 mg/mL) were first sonicated inside two vials for 5 min. Then, PDMS base at around 0.13 g/mL concentration was added into both solutions, which went through additional sonication for 1 h and mechanical stirring (magnetic stirrer at 1150 rpm) for 10 min. This process resulted in well dispersed solutions, as indicated in Figure 5a. However, due to the adverse effect of mechanical stirring on an established dispersion state which will be discussed in next section, dispersed solutions became unstable 4 h after stirring, as revealed in Figure 5b, leadings to visible phase separation in the pristine MWCNTs solutions. Relatively speaking, it was clear that the COOH-MWCNT dispersion, after going through the same processing steps, was much more stable than the pristine MWCNT dispersion. Therefore, it could be verified that carboxyl functionalized carbon nanotubes had better dispersion in polymers than pristine carbon nanotubes.

A potential reason for that was the covalent bond between CNTs and the polymer matrix due to surface functionalization could help prevent nanotubes from agglomerating and forming bundles. Moreover, it was thought that, aside from dispersion, the production of robust nanocomposite materials may prefer strong covalent chemical bonding between the nanofillers and the polymer matrix rather than the much weaker van der Waals physical bonds which occur if the fillers were not functionalized [27].

### 2.4. Comparison of Dispersion Approaches

Amongst the main forms of physical dispersion methods three are particularly interesting: shear mixing, mechanical stirring, and sonication. In effort to optimize the dispersion process in the preparation of polymer nanocomposite, in this work these three approaches were experimentally tested and compared in terms of their performance, and a combinatory approach was proposed for the fabrication process.

#### 2.4.1. Shear Mixing

Shear mixing separates individual CNTs from bundles via the shear flow induced by the rotation of an extrusion in a polymer solution or melt. Usually, dispersion via shear mixing is only achievable for specific types of MWCNTs, with high shear rate in a rather viscous medium. Therefore, it does not necessarily require any common solvent to assist dispersion, which simplifies the process. Nanocomposite containing high loadings of CNTs (up to 7 wt.%) have been realized via this technique [21]. However, a major downside is that the processing time significantly goes up as loading concentration rises. More importantly, shear mixing tends to section carbon nanotubes into shorter length scale, thereby reducing their conductivity significantly—an undesired attribute for nanocomposite intended for use as a sensor material.

In our experiments, various loading percentages of MWCNTs (~2–8 wt.%) were shear mixed with PDMS base polymer using a drilling machine (GMC 10-inch drill press stage, rpm 1000–1500 rpm). It was found that, although CNTs could be well dispersed within PDMS directly, the conductivity of the final nanocomposite was simply compromised too much for it to work effectively as a sensing material. The shortening of nanotubes due to shearing caused conductivity to decrease by more than 10 times compared to other approaches, which was undesirable for conductive nanocomposite. Therefore, this method was eventually not incorporated into the process of nanocomposite fabrication.

#### 2.4.2. Sonication

Being the most popular dispersion technique today, ultrasonic agitation exposes CNTs to ultrasonic waves and transfers shear forces to individual nanotubes which break them from agglomerates. There are two frequencies of ultrasonic waves that are used: (1) low frequency (~20–24 kHz); and (2) high frequency (~42–50 kHz). The sonication bath used in this work has high frequency but relatively low power (FS20D Fisher Scientific, frequency 42 kHz, output power 70 W). Although higher power of the sonication bath (>500 W) is desired as it provides higher shearing force to break down CNT bundles, nevertheless, prolonged exposure could also cause damaging of CNTs (especially shortening) which could significantly decrease the conductivity of CNTs [28]. Considering all factors, a mild sonication bath would be an ideal option as it allowed high quality of dispersion yet avoided severe damaging of CNTs during sonication.

Sonication was an essential process to control because it covered most of the nanocomposite preparation steps. Temperature was an important factor to control and normally needed to be low in the initial dispersion stage. Due to lack of cooling sinks on sonication bath, temperature was maintained relatively constant by changing bath water about every 30 min.

#### 2.4.3 Mechanical Stirring

Mechanical stirring facilitates dispersion as it creates shearing force through the high speed rotary motion of the stirrer. In this work, a magnetic stirrer was used which could rotate at maximal of 1150 rpm. Experiments found that, for the dissolution of PDMS in organic solvents, magnetic stirring worked more efficiently time wise than sonication. To dissolve 2 g of PDMS base in 15 mL of chloroform, for example, magnetic stirring (1150 rpm) could shorten dissolution time (could be estimated by disappearance of phase separation) from 1 h (sonication time) to around 8 min which was a significant improvement.

In our experiments, however, mechanical stirring was found to be not beneficial for the improvement of CNT dispersion quality. As a matter of fact, it was shown to have an adverse effect on the dispersion state of PDMS-MWCNTs in common solvents. For the pristine MWCNT solution shown in Figure 5 above, the PDMS-MWCNTs dispersion in chloroform was, with 1 h of sonication, stable when observed 3 days afterwards. But when solution went through 10 additional minutes of mechanical stirring (1150 rpm), the dispersion almost immediately became less stable, showing visible CNT agglomerates in the vial.

After comparing the three common dispersion approaches, it seemed that a combinatory approach would provide the optimal process. Mechanical stirring could be used for the initial dissolution of PDMS base in common solvent, as it was more time efficient. Sonication, on the other hand, could be used in the other aspects/steps of material preparation.

### 2.5. Experimental Procedure for Preparation of Polymer Nanocomposite

After numerous experimental trials and improvements, the so-far optimized procedure to obtain homogenous polymer nanocomposite is as follows.

In the stage of initial dispersion, first COOH-MWCNTs (e.g., 0.2 g) are added into a solution of chloroform (e.g., 50 mL) inside a metric cylinder (e.g., 100 mL). Note that the CNT concentration here (4 mg/mL) is much higher than that used in the testing section, perhaps even higher than the solubility of CNTs in chloroform, but it was necessary to have a relatively high concentration because the weight of final nanocomposite needs to be at least a few grams to be useable. Plus, the small diameter of cylinder is usually preferred over a wide mouse beaker, because as little as the final polymer nanocomposite is (e.g., for 5 wt.% PDMS-CNTs, 0.2 g MWCNTs could produce only 4 g final nanocomposite), a wide beaker would cause majority of nanocomposite to stick onto the wall and bottom areas, leaving little for later usage.

After initial mixing of COOH-MWCNTs and chloroform, the mixture is sonicated for around 1 h. Meanwhile, PDMS base resin (e.g., 3.5 g) is added into a separate solution of chloroform (e.g., 10 mL), and stirred with a magnetic stirrer (1150 rpm) for 15 min. Then, the two solutions are mixed together to go through 1–2 h of additional sonication to ensure sufficiently uniform dispersion of MWCNTs and PDMS. Experiments suggest that, on top of this time, further extended time of sonication does not significantly improve dispersion quality anymore.

Next in the state of solvent evaporation, it is highly desirable to minimize the required time to fully dry up the organic solution. As the concentration of CNTs continuously rise (for more than 2 orders) during the solvent drying process, some reaggregation of nanotubes is bound to happen. To minimize the size and amount of CNT agglomerates, solution should be dried as soon as possible. In this work, two techniques have been introduced to help expedite the solution evaporation process.

Firstly, the temperature of the nanocomposite-containing chloroform solution could be raised close to its boiling point (61.2 °C). At this temperature, the properties of the nanocomposite stay virtually intact while the drying process dramatically speeds up. Simply, the fastest way to elevate solution temperature is to pour in pre-boiled water into the sonication bath, and carefully mix it to adjust the temperature to be at or slightly over the boiling point.

Secondly, the introduction of a vacuum pump into the cylinder could speed up the evaporation process as well. One problem with the narrow-mouthed cylinder is that it usually takes days for solution (e.g., 50 mL) to fully evaporate even at elevated temperature, since vapor molecules get saturated inside cylinder and could not quickly escape. A Teflon tube connected to a vacuum pump could quickly remove the chloroform vapors from the upper portion of the cylinder, thus reducing evaporation time significantly from several days to a couple of hours (actual time depends on solution volume, CNTs percentage, temperature and vacuum level).

Finally, after the complete evaporation of the common solvent and before the microfabrication of polymer nanocomposite, curing agent—another part of PDMS polymer should be introduced into the mixture. Since the mixing of curing agent and base polymer would cause PDMS to gradually solidify, usually in 24 h at room temperature, it is desired to minimize the mixing time for curing agent. Simple manual mixing for 10–20 min is normally carried at here. At the mixing ratio of 10:1 (base to curing agent ratio), experiments suggest that the relatively short manual mixing time does not alter the dispersion quality obviously.

### 2.6. Dispersion Characterization of Final Nanocomposite

While the dispersion and clustering of spherical particles has been studied well, for both spherical and highly asymmetrical (platelets, rods and fibers) [29,30,31,32], it has remained a technical challenge to directly and reliably observe carbon nanotubes in the bulk of a nanocomposite suspension. All optical methods (e.g., optical microscopy) cut off below a length scale of 0.2–0.5 μm; all electron microscopy methods, though prominent in observations of individual nanotubes, could only provide information about the sample surface, *i.e.*, only representative for the selected fields of view. This leaves reciprocal space techniques and, more importantly, global indirect techniques of characterizing the dispersed nanocomposites; each of these techniques suffers from the unavoidable difficulty in interpretation of results.

Although attempts have been made to quantitatively assess the dispersion characteristics of CNTs inside polymer matrices (e.g., using Minkowski connectivity, radial power spectral density) [33], most reports still have to rely on optical and electron micrographs, despite their shortcomings, to evaluate relative quality of CNT dispersion [22,24,25,34].

**Figure 6:**
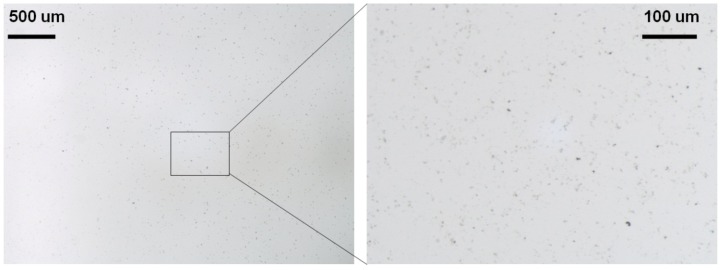
Optical micrographs of CNT dispersion inside a solution which contains chloroform, PDMS and functionalized MWCNTs.

In an effort to compare relative quality of various CNT dispersions within polymer matrices, this work has also adopted optical microscopy and electron microscopy for observation of CNT dispersion within polymer matrix. For instance, during the dispersion process, a drop of chloroform solution containing dispersed functionalized MWCNTs and PDMS base (CNT~4 mg/mL) was observed under a stereomicroscope. The optical micrographs shown in Figure 6 suggest that CNT cluster sizes generally did not exceed 10 μm. Compared to cluster sizes reported in existing literatures [22,34], this was indicative of relatively high dispersion quality.

**Figure 7:**
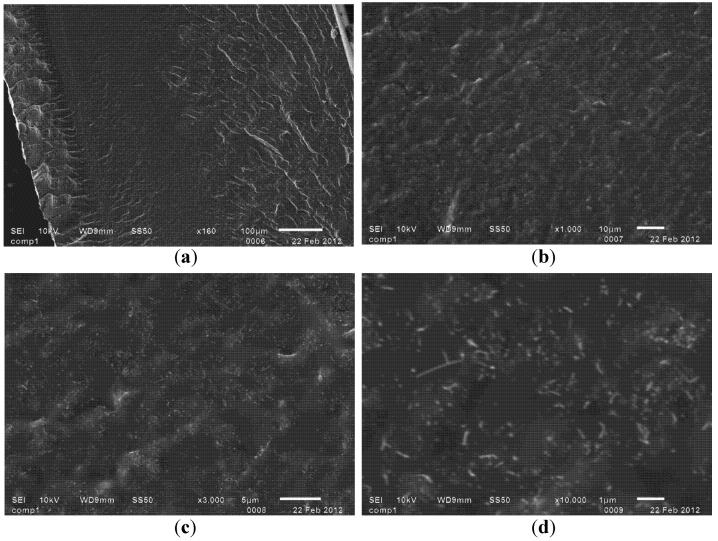
SEM images showing dispersed MWCNTs on a cross section of polymer nanocomposite that was fractured in liquid nitrogen. Nanocomposite contains around 7 wt.% of functionalized carbon nanotubes throughout its matrix. Observation was made on same area of surface with following increasing magnification: (**a**) 160×; (**b**) 1000×; (**c**) 3000×; and (**d**) 10,000×.

In the final polymer nanocomposite, it is difficult to use optical microscopy to directly observe the bulk dispersion of CNTs within polymer matrix, especially for those nanocomposite having a high loading percentage (>1%) of CNTs, since the samples normally become optically non-transparent. Scanning electron microscopy (SEM), on the other hand, provides a tool for observing the inside of a bulk sample. For example, a PDMS-MWCNTs nanocomposite sample containing functionalized MWCNTs was fractured in liquid nitrogen to obtain a cross section, and viewed under SEM, as in Figure 7.

After optimization of our dispersion procedure, SEM images demonstrated relatively uniform distribution of nanotubes throughout the fractured surface. Cluster size throughout the nanocomposite remained consistently under about 3 µm. Compared with previously reported SEM images, this indicates an excellent dispersion quality, especially for nanocomposite containing high percentage of carbon nanotubes (>5 wt.%).

## 3. Conclusions

In this work, to assist the dispersion of MWCNTs inside PDMS matrix and realize a uniform distribution, a common solvent was selected amongst various tested organic solutions. Based on its high solubility for PDMS and MWCNTs respectively, and its ability to retain dispersed state of MWCNTs in presence of PDMS, chloroform was found to be an optimal choice as a common solvent. Also, the surface functionalization of CNTs by carboxyl groups was found to be beneficial for further improvement of dispersion quality.

Through extensive testing of a variety of widely used physical dispersion techniques such as shear mixing, mechanical stirring and sonication, a combinatory approach was developed in which mechanical stirring was used to facilitate the initial dissolution of PDMS inside common solvent, and mild sonication used to as a main tool to disperse MWCNTs within PDMS. Following the dispersion stage of MWCNTs and PDMS within the common solvent, the evaporation process was facilitated and expedited by use of vacuum pump and accurate control of elevated temperatures. Solution drying time was significantly shortened, and thereby initial dispersion quality was largely retained throughout solvent evaporation.

Even at high loading concentrations of CNTs within polymer, high quality dispersion of nanocomposite was achieved, which showed significant improvement over prior approaches. Dispersion quality was studied using various characterization tools such as optical microscopy and electron microscopy. With high quality dispersion of CNTs achieved in this work, the polymer nanocomposite may prove to be a desirable structural candidate for a variety of applications.
